# Reading Aloud and Solving Simple Arithmetic Calculation Intervention (Learning Therapy) Improves Inhibition, Verbal Episodic Memory, Focus Attention and Processing Speed in Healthy Elderly People: Evidence from a Randomized Controlled Trial

**DOI:** 10.3389/fnhum.2016.00217

**Published:** 2016-05-17

**Authors:** Rui Nouchi, Yasuyuki Taki, Hikaru Takeuchi, Takayuki Nozawa, Atsushi Sekiguchi, Ryuta Kawashima

**Affiliations:** ^1^Creative Interdisciplinary Research Division, Frontier Research Institute for Interdisciplinary Science (FRIS), Tohoku UniversitySendai, Japan; ^2^Human and Social Response Research Division, International Research Institute of Disaster Science, Tohoku UniversitySendai, Japan; ^3^Smart Ageing International Research Centre, Institute of Development, Aging and Cancer, Tohoku UniversitySendai, Japan; ^4^Department of Nuclear Medicine and Radiology, Institute of Development, Aging and Cancer, Tohoku UniversitySendai, Japan; ^5^Division of Medical Neuroimage Analysis, Department of Community Medical Supports, Tohoku Medical Megabank Organization, Tohoku UniversitySendai, Japan; ^6^Division of Developmental Cognitive Neuroscience, Institute of Development, Aging and Cancer, Tohoku UniversitySendai, Japan; ^7^Department of Ubiquitous Sensing, PreClinical Research Center, Institute of Development, Aging and Cancer, Tohoku UniversitySendai, Japan; ^8^Department of Adult Mental Health, National Institute of Mental Health, National Center of Neurology and PsychiatryTokyo, Japan; ^9^Department of Functional Brain Imaging, Institute of Development, Aging and Cancer, Tohoku UniversitySendai, Japan

**Keywords:** learning therapy, cognitive training, reading aloud, simple calculation, transfer effect

## Abstract

**Background**: Previous reports have described that simple cognitive training using reading aloud and solving simple arithmetic calculations, so-called “learning therapy”, can improve executive functions and processing speed in the older adults. Nevertheless, it is not well-known whether learning therapy improve a wide range of cognitive functions or not. We investigated the beneficial effects of learning therapy on various cognitive functions in healthy older adults.

**Methods**: We used a single-blinded intervention with two groups (learning therapy group: LT and waiting list control group: WL). Sixty-four elderly were randomly assigned to LT or WL. In LT, participants performed reading Japanese aloud and solving simple calculations training tasks for 6 months. WL did not participate in the intervention. We measured several cognitive functions before and after 6 months intervention periods.

**Results**: Compared to WL, results revealed that LT improved inhibition performance in executive functions (Stroop: LT (Mean = 3.88) vs. WL (Mean = 1.22), adjusted *p* = 0.013 and reverse Stroop LT (Mean = 3.22) vs. WL (Mean = 1.59), adjusted *p* = 0.015), verbal episodic memory (Logical Memory (LM): LT (Mean = 4.59) vs. WL (Mean = 2.47), adjusted *p* = 0.015), focus attention (D-CAT: LT (Mean = 2.09) vs. WL (Mean = −0.59), adjusted *p* = 0.010) and processing speed compared to the WL control group (digit symbol coding: LT (Mean = 5.00) vs. WL (Mean = 1.13), adjusted *p* = 0.015 and Symbol Search (SS): LT (Mean = 3.47) vs. WL (Mean = 1.81), adjusted *p* = 0.014).

**Discussion**: This randomized controlled trial (RCT) can be showed the benefit of LT on inhibition of executive functions, verbal episodic memory, focus attention and processing speed in healthy elderly people. Our results were discussed under overlapping hypothesis.

## Background

Cognitive function changes during one’s lifetime (Hedden and Gabrieli, 2004). Previous reports have described that older adults showed a decline in memory performance (Salthouse, 2003), attentional process (Yakhno et al., 2007), processing speed (Salthouse, 1996) and executive functions (Royall et al., 2004). Defining the term executive function is important. The term “executive function” is used as an umbrella for various complex cognitive processes and sub-processes (Elliott, 2003). Executive function includes the high-level cognitive processes that facilitate new ways of behaving and which optimize one’s approach to unfamiliar circumstances (Gilbert and Burgess, 2008). Three main components in executive functions are “updating (constant monitoring and rapid addition/deletion of working-memory contents), shifting (switching flexibly between tasks or mental sets) and inhibition (deliberate overriding of dominant or proponent responses)” (Miyake et al., 2000; Miyake and Friedman, 2012). As described herein, we use executive function as a collective term includes updating, shifting and inhibition.

Previous studies have demonstrated improvements of cognitive functions in older adults (Verhaeghen et al., 1992; Ball et al., 2002; Clare and Woods, 2004; Edwards et al., 2005; Carretti et al., 2007; Green and Bavelier, 2008; Lustig et al., 2009; Jean et al., 2010; Nouchi et al., 2012a; Ballesteros et al., 2014). Cognitive training is an intervention program that provides structured practice on tasks related to aspects of cognitive function. There are many types of cognitive training such as working memory training (Richmond et al., 2011), processing speed training (Edwards et al., 2005, 2009), memory strategic training (Verhaeghen et al., 1992; Mahncke et al., 2006a; Carretti et al., 2007) and brain training game (Nouchi et al., 2012b; Anguera et al., 2013; Lampit et al., 2014; Toril et al., 2014). Developing cognitive training and validating the evidence of cognitive training have been at the forefront of scientific efforts (Nouchi and Kawashima, 2014).

Recently, cognitive training using reading aloud and solving of simple arithmetic calculations, so-called learning therapy (LT), has been developed (Kawashima et al., 2005; Uchida and Kawashima, 2008; Nouchi et al., 2012b). We were motivated to develop the new cognitive training based on following reasons. First, a previous study suggested that cognitive training should be designed in accordance with neuro-scientific evidence (Papp et al., 2009). Consequently, training tasks of learning therapy are created based on recent neuroimaging findings. Learning therapy uses reading text aloud and making simple calculations. Neuroimaging studies show that reading sentences with a voice (Miura et al., 2003, 2005) and simple arithmetic operations (Kawashima et al., 2004; Arsalidou and Taylor, 2011) activated the frontal, temporal and parietal cortices. Second, psychological stress related cognitive training reduced the improvements of cognitive functions after cognitive training (McAvinue et al., 2013). We selected extremely simple and easy tasks to do during intervention periods. Additionally, the training tasks are more familiar activities for older people because reading and calculation are parts of everyday life. Moreover, the training tasks use only papers and pencils. Consequently, elderly people can comprehend and perform training tasks. Third, previous results of studies have suggested that multiple components in training tasks facilitate to improvement of cognitive functions (Stuss et al., 2007; Green and Bavelier, 2008). Reading aloud and solving arithmetic problems are accomplished using a combination of cognitive processes including memory, attention and executive functions (inhibition, shifting and updating; Nouchi et al., 2012b). Therefore, we prepared reading aloud and mathematical calculations tasks as training tasks of learning therapy. Learning therapy is expected to have greater effects than previous cognitive training because it alleviates the difficulties mentioned above.

An earlier report described that learning therapy improves executive functions, episodic memory and processing speed (Kawashima et al., 2005; Uchida and Kawashima, 2008; Yoshida et al., 2014). However, there is no study which investigates the positive effects of learning therapy on various cognitive functions (e.g., memory and attention) in elderly people. Previous studies did not measure any cognitive function except for executive functions and processing speed. It is necessary to test the effectiveness of learning therapy on diverse cognitive functions, because various cognitive functions are necessary to support our actions and behaviors in everyday life.

As we mentioned before, the previous studies using learning therapy showed only improvements of executive functions, episodic memory and processing speed (Kawashima et al., 2005; Uchida and Kawashima, 2008; Yoshida et al., 2014). It is still unclear whether or not learning therapy can facilitate performance of other cognitive domains such as episodic memory, short term memory, working memory, reading ability and attention. Thus, this study was designed to investigate whether or not learning therapy can improve diverse cognitive functions in elderly people. There were essential differences from previous studies. First, the current study was aimed to validate the previous finding using the different cognitive tests in the same domains such as executive functions and processing speed. For example, previous studies used: (1) Frontal Assessment Battery (FAB) as the executive functional measure (Kawashima et al., 2005; Uchida and Kawashima, 2008); (2) the digit symbol coding as the processing speed measure (Kawashima et al., 2005; Uchida and Kawashima, 2008); and (3) the word list as the episodic memory measure (Yoshida et al., 2014). On the other hand, we newly used Stroop and verbal fluency tests for the executive functional measure, Symbol Search (SS) for the processing speed andLogical Memory (LM) and face-name association memory for the episodic memory measure. Second, we measure other cognitive domains such as focus attention, short-term memory and working memory which did not measure in the previous articles. Thus, we can firstly investigate the beneficial effects of LT on a wide range of cognitive functions in one study.

To elucidate the beneficial effects of learning therapy on a wide range of cognitive function, we conducted single-blinded randomized controlled trials (RCT) using learning therapy. Testers who conducted psychological tests were blinded to the study hypothesis and the group membership (learning therapy group or not). To test the benefits of learning therapy, we assessed a broad range of cognitive functions. We measured inhibition and shifting of executive functions, verbal and facial episodic memory, short-term memory, working memory, reading ability, focus attention and processing speed.

Based on previous studies using cognitive training related to reading and mathematical calculations, we hypothesized that learning therapy would improve the inhibition performance in executive functional domain, verbal episodic memory in episodic memory domain, focus attention and processing speed in the healthy elderly people. Several reasons exist: (1) regarding the inhibition performance, a previous study (Uchida and Kawashima, 2008) found that performance of FAB (Dubois et al., 2000) which measured executive functions improved after learning therapy. Especially, the sub score (sensitivity to an interference task) of FAB was increased after learning therapy. The sensitivity to an interference task requiring behavioral self-regulation under verbal commands conflicts with sensory information. It may be highly correlated with the inhibition ability in executive functions. This task is similar to the Stroop task. Cognitive training using calculation (Takeuchi et al., 2011) and video game training (Nouchi et al., 2013), which included reading aloud and mental calculations, in young adults showed the improvements of the Stroop task. Consequently, we hypothesized that learning therapy can improve the inhibition performance measured by Stroop task performance; (2) for episodic memory, a previous study using working memory training, which included calculation training, demonstrated improvements of episodic memory (McAvinue et al., 2013). An earlier long-term intervention study using reading aloud and calculation revealed improvements of verbal episodic memory as measured by word lists (Yoshida et al., 2014). Consequently, we hypothesized that learning therapy would engender improvements of verbal episodic memory performance measured by LM test; (3) for focus attention, to solve mathematical calculations and reading sentences, it is necessary to examine the present information (focus attention) specifically. We assume that the focus attention ability would be improved by simple calculation and reading aloud tasks; and (4) for processing speed, our report of a previous study described improvements of processing speed measured by digit symbol coding (Uchida and Kawashima, 2008). Additionally, video game training using reading aloud and calculations also showed improvements of processing speed measured by SS (Nouchi et al., 2013). Based on the previous finding, we hypothesized that processing speed measured by SS and digit symbol coding. Therefore, we assumed that learning therapy engenders improvements of inhibition, verbal episodic memory, focus attention and processing speed compared to the control group.

## Methods

### Randomized Controlled Trial Design and Setting of this Trial

The following information was the same as our protocol article (Nouchi et al., 2012b). This study was a RCT. This study “conducted in Sendai city, Miyagi prefecture, Japan. Written informed consent to participate in the study was obtained from each participant based on the Declaration of Helsinki before enrolment. Informed consent were approved by the Ethics Committee of the Tohoku University Graduate School of Medicine (ref. 2011-153). This study was registered in the University Hospital Medical Information Network (UMIN) Clinical Trial Registry (UMIN000006998)” (Nouchi et al., 2012b).

**Figure 1:**
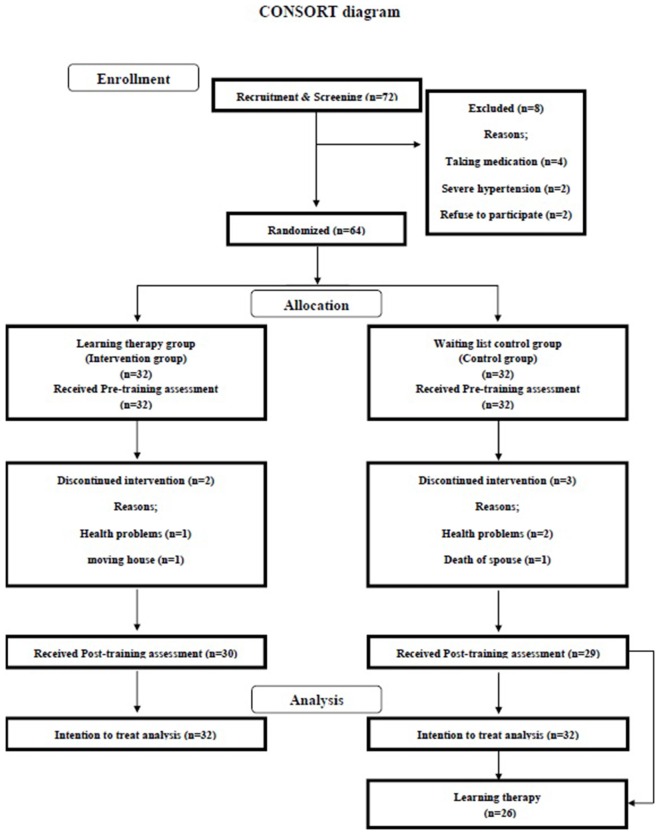
**Consolidated standards of reporting trials (CONSORT) flowchart**.

To assess the impact of learning therapy on widely diverse cognitive functions in healthy elderly people, we used a single-blinded intervention with two parallel groups: a learning therapy group and a waiting list (WL) control group. Testers were blind to the study’s hypothesis and the group membership of participants. The Consolidated Standards of Reporting Trials (CONSORT) statement (http://www.consort-statement.org/home/) was used as a framework for developing the study methodology (see Supplementary Material 1). The trial design is presented in Figure 1.

### Participants

Seventy-two participants were recruited from Sendai city through advertisements in the local town paper (Kahoku weekly; Figure 1). A procedure of recruitment of participants was the same as our previous study (Nouchi et al., 2014). Sample size calculation was written in Supplementary Material 2. Interested participants were screened using a semi-structured telephone interview, during which we asked participants for their demographic information (e.g., age and gender) and asked the same questions related to our inclusion and exclusion criteria (e.g., past medical histories of disease known to affect the central nervous system and using medications known to interfere with cognitive functions). “After the telephone interview, four people were excluded because they reported taking medications known to interfere with cognitive function (including benzodiazepines, antidepressants and other central nervous agents)” (Nouchi et al., 2012b). Two people were excluded based on severe hypertension (systolic blood pressure over 180, diastolic blood pressure over 110). Two participants declined to participate before a random assignment. All included participants (*n* = 64) were invited to visit Tohoku University for more detailed screening assessment (JART) and Mini-Mental State Examination (MMSE; Folstein et al., 1975) and to provide written informed consent. No participant was excluded based on JART scores. Randomization was conducted after receiving the informed consent statement from participants. Before the intervention period, we measured reading abilities using a sub-scale of Wechsler Adult Intelligence Scale (WAIS) such as Vocabulary (Wechsler, 1997) and arithmetic abilities using a sub-scale of WAIS such as Arithmetic (Wechsler, 1997). Table 1 presents the baseline characteristics. There were no significant differences between the groups in all data (two-sample *t*-test, *p* > 0.10). The scores of MMSE and IQ were within the range of normality.

**Table 1:** **Characteristics of participants in the learning therapy group and the waiting list control groups**.

	Learning therapy group		Wait list control group	
	Mean	SD	Mean	SD	*p*-value
Age (years)	72.81	(6.18)	71.38	(4.92)	0.31
Education (years)	13.25	(2.00)	12.69	(1.40)	0.20
MMSE (score)	28.34	(0.86)	28.44	(0.76)	0.65
Arithmetic from WAIS (score)	11.44	(2.42)	11.13	(2.42)	0.61
Vocabulary WAIS (score)	31.19	(4.37)	31.03	(4.65)	0.89
JART (score)	19.97	(3.47)	19.21	(4.30)	0.44
Estimated IQ from JART (score)	109.91	(7.03)	108.50	(8.61)	0.48

*No significant difference was found between combination exercise training and the waiting list control groups (two-sample t-test, p > 0.10). JART, Japanese Reading test; SD, standard deviation; MMSE, Mini-Mental State Examination; WAIS, Wechsler Adult Intelligence Scale III*.

Details of sample size calculation was described in the protocol of this study (Nouchi et al., 2012b).

### Learning Therapy Group (Cognitive Intervention Group)

**Figure 2:**
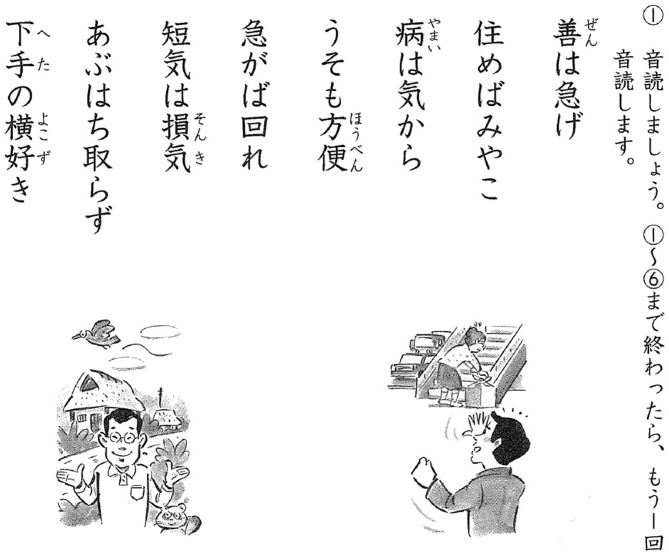
**An example of the training task for reading aloud**.

**Figure 3:**
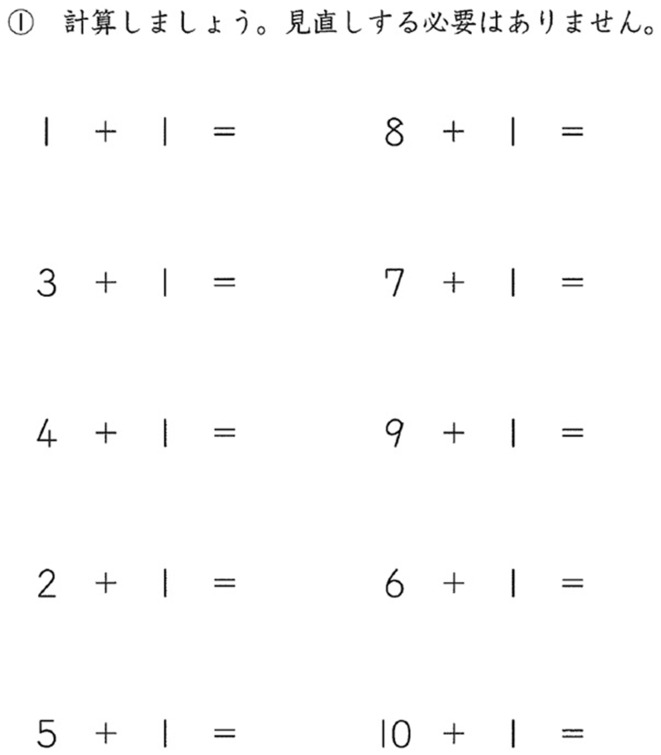
**An example of the training task for simple calculation**.

The cognitive intervention method was the same as that used in our previous study with learning therapy for healthy older adults (Uchida and Kawashima, 2008). Training tasks use two simple tasks (solving arithmetic and Japanese language problems) with systematized basic problems in arithmetic and reading (Uchida and Kawashima, 2008). The example of training tasks showed in Figures 2, 3. The lowest level of difficulty in simple calculation was simple addition (e.g., 1 + 3). The highest level of calculation was three figure number division (e.g., 156 ÷ 3). The lowest level of difficulty in reading aloud was reading simple sentences of 17 characters such as Japanese Haiku poems. The highest level was reading fairy tales aloud (about 100–120 characters per page). The difficulties of the Japanese Language problems were based on the number of Japanese characters per page because it takes more time to read aloud sentences with more characters.

Before intervention, we measured the percentages of correct answers and the time it takes to solve the diagnostic tests. The diagnostic tests consisted of 70 simple arithmetic and 16 simple reading tests. Based on the diagnostic tests, the appropriate level and workload in training tasks were set. In these tasks, participant was able to solve the problems with ease and without mental stress within 15 min. The difficulty level of training tasks and workloads did not change during the intervention period.

The cognitive intervention was scheduled to be conducted for 23 weeks. Participants in the cognitive intervention group were asked to go to a classroom at Tohoku University once a week. They were instructed to complete five sheets of each task prepared for each participant for that day. The daily learning time for the two tasks was approximately 15 min. They were required to complete five sheets of each task as homework for 4–6 days a week.

### Waiting List Control Group (No Cognitive Intervention Group)

The following texts were the same as our previous protocol article (Nouchi et al., 2012b). “The waiting list control group received no intervention. Those participants were informed by letter that they were scheduled to receive an invitation to participate after a waiting period of 6 months. No placebo was used for the social contact group” (Nouchi et al., 2012b). Results of previous intervention studies (Clark et al., 1997; Mahncke et al., 2006) showed that “a placebo group was unnecessary for study of this type because no difference existed in cognitive or functional improvement between the placebo and no-social-contact groups (control group)” (Nouchi et al., 2012b). After a waiting period of 6 months, 26 elderly people in the waiting list group participated in the learning therapy. Six elderly people declined to receive the learning therapy because three elderly people dropped out during the waiting period and three elderly did not want to receive the learning therapy after the waiting period.

There were several reasons why we used the waiting list control group. To investigate beneficial effects of cognitive training, we should use certain control conditions because of reductions in the influence of practice effects and cognitive declines by aging. Several studies used no-contact or waiting list groups (Levine et al., 2007; Basak et al., 2008; Shatil et al., 2010; McDougall and House, 2012). On the other hand, many activities were selected for active control conditions (Buiza et al., 2008; Peretz et al., 2011; Nouchi et al., 2012a). For instance, there were many types of activities in the active control groups such as selecting letters in the newspaper (Herrera et al., 2012), leaning trivia using web sites (Richmond et al., 2011a) and video games (Nouchi et al., 2012a). From a methodological perspective, it would be better to use an other intervention program as an active control group in the cognitive training studies compared to use a waiting list control group. However, to use an active control group, we should consider an ethical issue. Using the active control group should be “a reasonable balance of the level of benefit to the time investment required of participants in the treatment group” (Street and Luoma, 2002). An active control group with meaningless and worthless tasks is not suitable. In the cognitive training research field, there is no standard and appropriate task for the active control group. The choice of an active control condition must be considered within costs, research questions and ethical considerations. Additionally, results of previous intervention studies (Clark et al., 1997; Mahncke et al., 2006) reported that a placebo group was unnecessary for study of this type because no difference existed in cognitive or functional improvement between the placebo and no-social-contact groups (control group). Moreover, using the waiting list control group had “the advantage of letting everyone in the study receive the new intervention (sooner or later)” (Nouchi et al., 2014). Therefore, we decided to the waiting list control group.

### Cognitive Function Measures

The following texts were the same as our previous article (Nouchi et al., 2012b). To test the positive effects of learning therapy on performance in cognitive functions, we used several cognitive measures (Table 2). “Measures of the cognitive functions were divisible into seven categories (executive functions, episodic memory, short-term memory, working memory, reading ability, attention and processing speed)” (Nouchi et al., 2012b). Executive functions were measured by the Stroop Test (ST; Hakoda and Sasaki, 1990; Watanabe et al., 2011) and Verbal fluency task (VFT; Ito et al., 2004). The ST had ST and reverse ST (rST) conditions. For ST, participants were required to select a word corresponding to the ink color in the color–word combination. For rST participants were asked to choose a color item corresponding to a color–word combination’s semantic meaning or selection. For VFT, we used Japanese version’s letter fluency task (LFT) and category fluency (CFT) task. Participants were asked to generate many words beginning with the specific letter (LFT) or many words of a category (CFT). Episodic memory was measured using LM (Wechsler, 1987) and First and Second Names (FSN; Wilson et al., 1985). For LM, participants were asked to memorize the short story. For FSN, participants were asked to memorize FSN with faces. Verbal short-term memory was measured by Digit Span Forward (DS-F; Wechsler, 1997). For DS-F, participants were asked to memorize numbers and repeat the numbers. Working memory was measured using Digit Span Backward (DS-B; Wechsler, 1997). For DS-B, participants were require to memorize digit numbers and answer the number in reverse order. Reading ability was measured by the Japanese Reading Test (JART; Matsuoka et al., 2006). JART was reading test of 25 Japanese Kanji. The Digit Cancellation Task (D-CAT) was used to measure focus attention (Hatta et al., 2000). For D-CAT, participants were asked to check the target number in 12 rows of 50 digits. Digit Symbol Coding (Cd; Wechsler, 1997) and SS (Wechsler, 1997) were used for processing speed tests. For Cd, participants were required to write the symbol corresponding to the number. For SS, participants were required to search the target symbols in five symbols. Details of all tasks are described in the Supplementary Material 2. We assessed these cognitive function measures before and after the intervention period (6 months).

**Table 2:** **Summary of cognitive function measures**.

Cognitive function	Task
Executive functions	Verbal fluency task
	Stroop test
Attention	Digit cancellation task
Episodic memory	Logical memory
	First and second names
Short-term memory	Digit span forward
Working memory	Digit span backward
Reading ability	Japanese reading test
Processing speed	Digit symbol coding
	Symbol Search

### Analysis

This study was designed to evaluate the beneficial effect of learning therapy in elderly people. The following analysis method was also presented in our protocol article (Nouchi et al., 2012b). We calculated the change score (post-training score minus pre-training score) in all cognitive function measures. We conducted ANCOVA for the change scores in each cognitive test. The change scores were the dependent variable. Groups (learning therapy, waiting list control) were the independent variable. Pre-training scores in the dependent variable, sex and age were the covariates to exclude the possibility that any pre-existing difference of measure between groups affects the result of each measure and adjust for background characteristics. Significance was inferred for *p* < 0.05. We used Storey’s False discovery rate (FDR) correction methods to adjust the *p* values (Storey, 2002). Moreover, this report describes eta squared (*η*^2^) as an index of effect size (Cohen, 1988). Missing data were imputed using the expectation-maximization method, as implemented in the Statistical Package for the Social Sciences (SPSS) Missing Value Analysis. It imputed missing values using maximum likelihood estimation with observed data in an iterative process (Dempster et al., 1977; Nouchi et al., 2012b). All randomized participants were included in the analyses in line with their allocation, irrespective of how many sessions they completed (intention-to-treat principle). All analyses were conducted using software (SPSS ver. 18 or higher; SPSS Inc.).

## Results

Sixty-four elderly people participated in this RCT (learning therapy and waiting list control). Thirty of the 32 members of the learning therapy group and 29 of the 32 members of the waiting list control group were completed. We imputed missing values of two participants in the learning therapy group and three participants in the waiting list control group using intention-to-treat principle (see “Analysis” Section). The pre-training and post-training scores in cognitive functions are presented in Table 3.

**Table 3:** **Cognitive function scores before and after training period in both groups**.

	Learning therapy group				Wait list control group			
	Pre		Post		Pre		Post	
	Mean	SD	Mean	SD	Mean	SD	Mean	SD	*p*-value
Executive functions
LFT (number)	8.56	(2.53)	9.38	(2.64)	9.59	(3.35)	10.28	(2.82)	0.17
CFT (number)	11.06	(2.84)	11.72	(2.77)	11.41	(2.08)	11.44	(3.26)	0.58
rST (number)	27.44	(6.87)	31.31	(5.87)	28.63	(6.47)	29.84	(6.66)	0.48
ST (number)	41.41	(9.00)	44.63	(8.29)	40.78	(9.15)	42.38	(8.73)	0.78
Attention
D-CAT (number)	26.31	(5.87)	28.41	(4.98)	26.84	(5.48)	26.25	(5.47)	0.71
Episodic memory
LM (score)	7.31	(2.22)	11.91	(2.80)	8.09	(2.84)	10.56	(2.94)	0.23
FSN (score)	2.97	(1.60)	3.34	(1.89)	3.66	(1.79)	4.00	(2.06)	0.11
Short-term memory
DS-F (score)	5.13	(0.87)	4.97	(1.18)	5.56	(1.37)	5.34	(1.33)	0.16
Working memory
DS-B (score)	4.09	(0.86)	3.97	(0.82)	4.38	(1.29)	4.13	(1.13)	0.31
Processing speed
Cd (number)	64.56	(13.15)	69.56	(13.21)	66.84	(11.48)	67.97	(11.37)	0.61
SS (number)	33.00	(6.15)	36.47	(5.51)	32.81	(6.17)	34.63	(6.41)	0.90
Verbal ability
JART (score)	19.97	(3.47)	20.34	(3.30)	19.25	(4.30)	19.50	(4.15)	0.46

*Group comparison (two sample t-tests) of the pre-training scores revealed no significant difference in any measure of cognitive functions between the learning therapy group and the waiting list control group (p > 0.10). Pre, pre-training; post, post-training; SD, standard deviation. Executive functions were measured using the Stroop Test (ST) and Verbal fluency task (VFT). Attention was measured using the Digit Cancellation Task (D-CAT). Episodic Memory was measured using Logical Memory (LM) and First and Second Names (FSN). Short-term memory was measured using Digit Span Forward (DS-F). Working memory was measured using Digit span Backward (DS-B). Processing speed was measured using Digit Symbol Coding (Cd) and Symbol Search (SS). Reading ability was measured using the Japanese Reading Test (JART)*.

To test the positive effect of the learning therapy on the improvement of cognitive functions, we did ANCOVA for the change scores in each cognitive test (Table 4). We found significant group differences in four cognitive domains (executive functions, episodic memory, attention and processing speed). For executive functions, the learning therapy group improved the ST score (learning therapy (Mean = 3.88) vs. waiting list control (Mean = 1.22), *F*_(1,59)_ = 7.35, *η*^2^ = 0.11, *adjusted p* = 0.013) and the rST score (learning therapy (Mean = 3.22) vs. waiting list control (Mean = 1.59), *F*_(1,59)_ = 8.72, *η*^2^ = 0.09, *adjusted p* = 0.015). For episodic memory, the learning therapy group improved the LM score (learning therapy (Mean = 4.59) vs. waiting list control (Mean = 2.47), *F*_(1,59)_ = 9.72, *η*^2^ = 0.12, *adjusted p* = 0.015). For focus attention, learning therapy group improved the D-CAT score (learning therapy (Mean = 2.09) vs. waiting list control (Mean = -0.59), *F*_(1,59)_ = 12.23, *η*^2^ = 0.14, *adjusted p* = 0.010). For processing speed, learning therapy group improved the Cd score (learning therapy (Mean = 5.00) vs. waiting list control (Mean = 1.13), *F*_(1,59)_ = 9.85, *η*^2^ = 0.13, *adjusted p* = 0.015) and the SS score (learning therapy (Mean = 3.47) vs. waiting list control (Mean = 1.81), *F*_(1,59)_ = 7.81, *η*^2^ = 0.10, *adjusted p* = 0.014). These results demonstrate that the learning therapy led to improvements of inhibition performance in executive functions, verbal episodic memory’s measures, focus attentional measure and all processing speed’s measures.

**Table 4:** **Change scores in cognitive functions measures of both groups**.

	Learning therapy		Wait list control	
	Mean	SD	Mean	SD	Effect size (*η*^2^)	*p*-value	*Adjusted* *p*-value	Results of ANCOVA
Executive functions
LFT (number)	0.81	(1.82)	0.69	(2.21)	0.00	0.607	0.759	ns
CFT (number)	0.66	(2.59)	0.03	(2.79)	0.01	0.396	0.566	ns
rST (number)	3.22	(1.90)	1.59	(2.53)	0.09	0.009	0.015	LT > WL
ST (number)	3.88	(3.44)	1.22	(3.90)	0.11	0.005	0.013	LT > WL
Episodic memory
LM (score)	4.59	(2.75)	2.47	(2.30)	0.12	0.003	0.015	LT > WL
FSN (score)	0.38	(1.79)	0.34	(1.81)	0.00	0.650	0.650	ns
Short-term memory
DS-F (score)	(0.16)	(1.05)	(0.22)	(0.83)	0.01	0.457	0.784	ns
Working memory
DS-B (score)	(0.13)	(0.75)	(0.25)	(1.34)	0.00	0.862	0.781	ns
Attention
D-CAT (number)	2.09	(2.99)	(0.59)	(3.15)	0.14	0.001	0.010	LT > WL
Processing speed
Cd (number)	5.00	(4.31)	1.13	(4.77)	0.13	0.003	0.015	LT > WL
SS (number)	3.47	(2.98)	1.81	(2.60)	0.10	0.007	0.014	LT > WL
Verbal ability
JART (score)	0.38	(1.50)	0.25	(1.30)	0.00	0.622	0.691	ns

*Change scores were calculated by subtracting the pre-cognitive measure score from the post-cognitive measure score. We conducted analysis of covariance (ANCOVA) for the change scores in each of cognitive tests. In the ANCOVA, pre-training scores in each cognitive test, sex and age were the covariates. Significance was inferred for *p* < 0.05. The *p* values were adjusted using FDR method. Moreover, this report describes eta square (*η*^2^) as an index of effect size. As a descriptive index of strength of association between an experimental factor (main effect or interaction effect) and a dependent variable, *η*^2^ is defined as the proportion of total variation attributable to the factor, and it ranges in value from 0 to 1. *η*^2^ ≥ 0.01 is regarded as a small effect, *η*^2^ ≥ 0.06 as a medium effect, and *η*^2^ ≥ 0.14 as a large effect. LT, learning therapy; WL, waiting list control group; SD, standard deviation. Executive functions were measured using the Stroop Test (ST) and Verbal fluency task (VFT). Episodic Memory was measured using Logical Memory (LM) and First and Second Names (FSN). Short-term memory was measured using Digit Span Forward (DS-F). Working memory was measured using Digit span Backward (DS-B). Attention was measured using the Digit Cancellation Task (D-CAT). Processing speed was measured using Digit Symbol Coding (Cd) and Symbol Search (SS). Reading ability was measured using the Japanese Reading Test (JART)*.

## Discussion

We investigated the positive effects of learning therapy on diverse cognitive functions in healthy elderly people. Results showed clearly that the learning therapy group exhibited improved inhibition performance of executive functions measured by ST and rST, verbal episodic memory measured by LM, focus attention measured by D-CAT and processing speed measured by SS and Cd compared to the waiting list control group. These results supported our hypothesis. The improvements of Cd performance of processing speed after learning therapy are consistent with previous evidence (Kawashima et al., 2005; Uchida and Kawashima, 2008). However, this study extends the previous finding by demonstrating improvements of focus attention and by replicating improvement of inhibition process and processing speed using different cognitive functional measures such as ST, rST and SS after learning therapy.

The overlapping hypothesis can explain the present results (Nouchi et al., 2012a, 2013, 2014). The overlapping hypothesis hypothesizes that improvements of cognitive functions by a certain type of training would occur if the processes during both cognitive training tasks (e.g., reading aloud and calculation simple arithmetic problem) and untrained tasks (e.g., measures of cognitive functions) were overlapped and were involved in similar cognitive processes. In this study, participants were required to undertake learning therapy, which included a reading aloud task and a simple arithmetic calculation problem task. As we described in the background reading aloud and simple arithmetic calculation involve many cognitive components and processes. To perform these tasks, inhibition process related to executive functions, verbal episodic memory, focus attention and processing speed are expected to be recruited. For instance, attentions are necessary to modulate one’s own voice during reading sentences aloud and inhibition ability is important to select and use rules of mathematics during calculation. Long-term Memory (semantic and verbal episodic memory) is expected to be necessary to read a sentence and to comprehend the sentence during reading aloud (LaBerge and Samuels, 1974; Myers et al., 2000). When we read a sentence, we construct a mental representation, known as the situation model (Kintsch, 1994; Zwaan and Radvansky, 1998). To facilitate understanding and integration of incoming information from sentence, we use related information from verbal long-term memory (Cook et al., 1998). Both semantic and verbal episodic memories are important to create and elaborate the situation model (Van Dijk and Kintsch, 1983; Kintsch et al., 1999). Some previous studies showed that semantic memory has the most important role in reading and comprehending the sentences (Garrod and Terras, 2000). Especially, the patient with episodic memory dysfunctions has no problem with reading (Tulving et al., 1988). Thus, most theories assume that comprehending and reading a sentence rely on more on semantic rather than on episodic memory. However, for healthy people, verbal episodic memory may influence reading processing as early as general world knowledge (Myers et al., 2000). One previous study reported that the verbal episodic memory for prior text facilitated the reading process (Haviland and Clark, 1974). Verbal episodic memory for prior texts is reactivated automatically when we encounter the related information during reading sentences (Myers and O’Brien, 1998). Consequently, verbal episodic memory is also necessary to read and comprehend texts as well as semantic memory. Focus attention is expected to be necessary to perform each task with concentration. Processing speed has a important role to solve mathematical problems and to read as fast as possible during a training period. The overlapping hypothesis explains the improvements of inhibition in executive functions, verbal episodic memory, focus attention and processing speed after learning therapy that follows. First, it would take the mental processes described above to perform the reading aloud task and the simple arithmetic calculation problem task. Second, both these training tasks and the cognitive tests can share the similar cognitive processes. Third, the cognitive processes described above are expected to be facilitated by these training tasks. Therefore, inhibition performance in executive functions, verbal episodic memory, focus attention and processing speed were improved after these training tasks.

Considering common elements of improved cognitive functional measures, it may be true that learning therapy generally improved the speed of processing related to task performance. Improved cognitive functional measures such as ST, rST, D-CAT, SS and Cd required participants to do the task as quickly as possible during a limited time (please see “Methods” Section). Two training tasks also require that participants complete materials of two training tasks as quickly as possible. Based on the overlapping hypothesis, both cognitive training tasks such as reading aloud and simple calculations and the psychological tests which measured the cognitive functions can share the elements of speed of processing. Therefore, performances of cognitive functional measures that require speed of participants were improved by learning therapy.

It is important to consider dissociations of improvements in executive functions and episodic memory measures. The benefits of learning therapy did not generalize to all tasks in the same functions. In this study, the learning therapy group improved inhibition performance of executive functions (rST and ST) but not other shifting performance of executive functions (LFT and CFT). Additionally, the learning therapy improved verbal episodic memory measure such as LM, but not the facial episodic memory measure such as FSN. According to the overlapping hypothesis, the possibility exists that these training tasks (reading aloud and calculation simple arithmetic problems) and cognitive function measures such as LFT, CFT and FSN might not share the same cognitive processes. For instance, previous studies showed that LFT and CFT require both the generation of words within a subcategory (clustering) and the ability to shift to a new category when a subcategory is exhausted (switching; Troyer et al., 1998). FSN requires a face recognition process and an association between FSN and between faces and names (Wilson et al., 1985). As described earlier herein, these cognitive processes (clustering, switching, face recognition and association) did not include cognitive processes during reading aloud and arithmetic calculation problems. Consequently, the respective levels of performance of LFT, CFT and FSN were not improved by learning therapy.

Dissociation result of episodic memory can also be explained by the following ideas. For episodic memory, participants may become more familiar with the story structure by reading aloud training. Therefore, participants would be able to use the knowledge to encode information better after the reading aloud training. This idea can predict only the improvement of LM, but not the FSN.

From the perspective of definition of executive functions, three main components exist in executive functions. Based on this, learning therapy improved only the inhibition ability in executive functions, but not the shifting ability. The improvement of the inhibition ability after reading aloud and simple calculation training was consistent with our previous results (Uchida and Kawashima, 2008; Nouchi et al., 2013). Our dissociation results of executive functions suggest that it is necessary to prepare multiple cognitive measures in each cognitive domain such as executive functions and episodic memory. Moreover, in future studies, we have to focus on one area of cognitive domain (e.g., working memory) and use varied measures for the specific cognitive domain (operating span, reading span, n-back and digit (spatial) span). It may reveal a great deal about the impact of cognitive intervention on cognitive functions in the elderly people.

In addition, discussion of negative findings of no improvements of verbal ability, short-term memory and working memory is important. For verbal ability measured by JART, participants must read Kanji compound words. The difficulty level of the kanji words in JART is higher than that in the junior high school. However, the difficulty level of kanji in the reading aloud task would be lower than that in JART because our texts for the reading aloud task are based on first-grade to fourth-grade elementary school students (please see “Methods” Section). Thus, the participants did not learn how to read difficult kanji through intervention. Therefore, learning therapy did not show the improvement of JART performance. In addition, we found no significant improvements of short-term and working memory measured by DS-F and DS-B. No improvements of short-term and working memory performance would come from the lack of adaptive elements for the training tasks. Some previous studies demonstrated that intensive adaptive training would facilitate improvements of cognitive functions (Takeuchi et al., 2011; McAvinue et al., 2013). One previous study using mental calculation training in young adults reported that the intensive adaptive training using mental calculation led to improvement of short-term memory performance, but not the no intention adaptive training using mental calculation (Takeuchi et al., 2011). In the present study, we set the appropriate difficulties level of both task based on the diagnostic tests. However, the difficulty level of both training tasks did not change through intervention periods. Thus, learning therapy showed no improvement of short-term and working memory performance. Future studies demand additional detailed studies to ascertain whether learning therapy using the intensive adaptive training would improve short-term and working memory performance.

This study has several strength points compared to earlier studies assessing the effects of cognitive training for elderly people. As we described in our protocol article (Nouchi et al., 2012b), we used easy to learn training tasks such as reading sentence and simple calculation using a paper and pencil. Most training tasks in previous studies were complex tasks using computers (Berry et al., 2010; Li et al., 2010). Using computers might make it easy to record data precisely and to control tasks. Nevertheless, elderly people often have difficulty using computers (Wagner et al., 2010). Recent studies have demonstrated that some barriers to computer use exist, such as lack of interest, lack of knowledge and fear of using computer (Saunders, 2004; Peacock and Künemund, 2007). The difficulty of using computers is one main reason of frustration and other negative emotions, possibly reducing their motivation to continue. On the other hand, our training tasks are easy to do for elderly people. Therefore our training tasks were expected to encourage their willingness.

This study has a limitation. We do not use other cognitive training as an active control group. We used the waiting list control group because previous studies revealed no differences in cognitive or functional improvement between the active control group and the non-training control group (Clark et al., 1997; Mahncke et al., 2006). However, from a study design perspective, it would be suitable to use the active control group to prove the effectiveness of learning therapy. Future intervention studies should be conducted to compare learning therapy to other cognitive training such as working memory training or training programs of other types such as exercise training.

## Conclusion

In summary, this study was designed to investigate the beneficial effects of learning therapy on diverse cognitive functions in healthy elderly people. This report is the first of a study revealing the beneficial effects of learning therapy on inhibition performance of executive functions, verbal episodic memory, focus attention and processing speed in healthy elderly people. On the other hand, the learning therapy did not improve shifting performance of executive function, facial episodic memory, verbal short-term memory, verbal working memory and reading ability. These positive and negative findings would be important for people who want to use the learning therapy. If people want to improve inhibition performance, verbal episodic memory, focus attention and processing speed, this intervention program would work well. If people want to improve other cognitive domains (e.g., working memory), other cognitive training program such as working memory training would be well for them. This study had some limitations, but produced important and sufficient evidence demonstrating the effectiveness of learning therapy. Given that cognitive functions are important to do daily life activities (Cahn-Weiner et al., 2000, 2002; Lee et al., 2005), our results suggest that cognitive functions in the elderly are enhanced through daily life activities such as reading aloud and calculating simple arithmetic problems.

## Author Contributions

RN designed, developed the study protocol and calculated the sample size. RN conducted the study. RN wrote the manuscript with YT, HT, TN, AS and RK. RK also provided advice related to the study protocol. All authors read and approved the final manuscript.

## Conflict of Interest Statement

Learning therapy was developed by RK and the Kumon Institute of Education. However, RK derives no income from Kumon Institute of Education and Society for Learning Therapy. RK has no other competing interests. All other authors have declared no competing interests.
