# Supplementary material for: Reading Aloud and Solving Simple Arithmetic Calculation Intervention (Learning Therapy) Improves Inhibition, Verbal Episodic Memory, Focus Attention and Processing Speed in Healthy Elderly People: Evidence from a Randomized Controlled Trial
Source: Front Hum Neurosci. 2016 May 17;10:217. doi: 10.3389/fnhum.2016.00217 (PMC4868921; doi:10.3389/fnhum.2016.00217)
Supplement: Supplementary file 2 [file Presentation_1.pdf]

Supplementary Material

Reading aloud and solving simple arithmetic calculation intervention (Learning therapy) improves inhibition, verbal episodic memory, focus attention, and processing speed in healthy elderly people: Evidence from a randomized controlled trial

Rui Nouchi*, Yasuyuki Taki, Hikaru Takeuchi, Takayuki Nozawa, Atsushi Sekiguchi, Ryuta Kawashima

*** Correspondence:** Corresponding Author: rui.nouchi.a4@tohoku.ac.jp

Sample Size

Our sample size estimation was based on the change score in the reverse Stroop task, which is the primary outcome in this study. The following text was also presented in our protocol paper [1]. We selected ST as the primary outcome measure because 1) learning therapy was expected to improve executive functions, and a previous study showed that learning therapy can improve executive functions, as measured by FAB [2], 2) reverse ST is a task that is often used to measure executive functions [3], and 3) reverse ST has been standardized, with high reliability and validity in Japanese

populations [4, 5].

We expected to detect a large effect size (η^2= 0.14) of the change score in the reverse ST between learning therapy and waiting list control groups. the sample size was determined using G * power [6] based on 80% power, a two-sided hypothesis test, an alpha level of 5%, an analysis of covariance (ANCOVA) model that includes a baseline reverse ST, age and sex as a covariate. The sample size calculation indicated that we need 32 participants in each of the learning therapy and waiting list control groups with consideration of a 20% drop-out rate.

Inclusion and Exclusion Criteria

This intervention was conducted to investigate the beneficial effects of learning therapy for a range of cognitive functions in healthy older adults. Details of inclusion and exclusion criteria were also presented in our protocol paper [1]. The criteria included participants who reported themselves to be right-handed, native Japanese speakers, unconcerned about their own memory functions, not using medications known to interfere with cognitive functions (including benzodiazepines, antidepressants or other central nervous agents), and having no disease known to affect the central nervous system, including thyroid disease, multiple sclerosis, Parkinson disease, stroke, severe

hypertension (systolic blood pressure over 180, diastolic blood pressure over 110), and diabetes. All subjects had normal vision. None had a history of neurological or psychiatric illness. Each participant was over 65 years old. Criteria excluded participants who had Intelligence Quotient (IQ) lower than 85, as derived from Japanese Reading Test (JART) [7]. Participants who had participated in another cognition-related intervention study were excluded.

Details of cognitive functional measures

ST: Stroop task (ST) measured executive functions including response inhibition and impulsivity. Hakoda's version was a paper and pencil version of ST [4]. In this test, participants must check whether their chosen answers were correct, unlike the traditional oral naming ST. We used a reverse ST and a ST. In the reverse ST, in the leftmost of six columns, a word naming a color was printed in another color (e.g., ‘red’ is printed in blue letters); the other five columns were filled respectively with five different colors from which participants must check the column with the color matching the written word in the leftmost column. In the ST, in the leftmost of six columns, a word naming a color was printed in another color (e.g., ‘red’ is printed in blue letters) and the other five columns contain words naming colors. Participants must check the

column containing the word naming the color of the word in the leftmost column. In each task, participants were instructed to complete as many of these exercises as possible in 1 min. The primary measure for this task is the number of correct items.

VFT: Verbal fluency task (VFT) measured executive functions. We used the Japanese Version of VFT [8], which had two tasks (letter fluency task (LFT) and category fluency (CFT) task). In LFT, a Japanese letter, 'ka', was given to each participant, who was then asked to generate common nouns beginning with this letter—as many as possible in 60 s. In CFT, a category name (Animal) was given to each participant, who was then asked to generate many words of a certain category (Animal). The participants were instructed not to include proper nouns or to repeat one that had already been stated. The primary measure for this task was the number of reported words. The reliability and validity of Japanese LFT were independently verified [8].

LM: Logical Memory (LM) evaluated the performance of episodic memory. LM was a subtest of the Wechsler Memory Scale-Revised (WMS-R) [9]. LM consisted of two short-paragraph-length stories (Story A and Story B). In LM, participants must memorize the short story. The stories were scored in terms of the number of story units recalled, as specified in the WMS-R scoring protocol. We used either Story A or Story B. The primary measure for this task was the number of correct story units recalled.

FSN: First and Second Names (FSN) evaluated memory ability in everyday life. FSN was a subset of Rivermead Behavioral Memory Test (RBMT) [10]. RBMT measured episodic memory as it was used in everyday life. Therefore, subsets of RBMT were similar to everyday situations. In FSN, participants must memorize first and second names with faces (photographs). Subsequently, they must recall the first and the second names when the face was shown again later. We used four faces (four first names and four second names). The primary measure of this test was the total number of correct answers in both first and second names. The maximum raw score of FSN was 8.

***DS*:** Digit Span (DS) was a subtest in Wechsler Adult Intelligence Scale-Third Edition (WAIS-III) [11]. Digit Span, which had two subsections (DS-F and DS-B), evaluated short-term memory and working memory. DS-F measured short-term memory by simply requiring participants to repeat numbers. DS-B measured working memory by requiring participants to memorize numbers and repeat the numbers in reverse order. For DS-F, participants repeated numbers in the same order as they were read aloud by the examiner. For DS-B, participants repeated numbers in the reverse order of that presented aloud by the examiner. In both, the examiner read a series of number sequences which the examinee must repeat in either forward or reverse order. DS-F has 16 sequences. DS-B had 14 sequences. The primary measures of this test were raw

scores that reflect the number of correctly repeated sequences until the discontinue criterion (i.e., failure to reproduce two sequences of equal length) was met [11]. The maximum raw score of DS-F was 16. The maximum raw score of DS-B was 14.

JART**:** The Japanese Reading Test (JART) measured reading ability [12]. JART was a Japanese version of the National Adult Reading Test (NART), which had a reading test of 50 irregularly spelled words in English (e.g. ache) [13]. JART was a reading test comprising 25 Kanji compound words (e.g. 親父, 煙草). The reading stimuli were printed out randomly for reading. The participants were asked to read each Kanji compound word aloud. This task assessed reading ability and IQ. The primary measure for this task was the number of correct items.

D-CAT**:** The Digit Cancellation Task **(**D-CAT) evaluated attention [14]. The test sheet consisted of 12 rows of 50 digits. Each row contains five sets of numbers 0–9 arranged in random order. Consequently, any one digit appeared five times in each row with randomly determined neighbors. D-CAT consisted of three such sheets. Participants were instructed to search for the target number(s) that had been specified to them and to delete each one with a slash mark as quickly and as accurately as possible until the experimenter sends a stop signal. Three trials were done, first with a single target number (6), second with two target numbers (9 and 4), and third with three (8, 3, and 7).

Each trial was administered for 1 min. Consequently, the total time required for D-CAT was 3 min. In the second and third trials, it was emphasized that all the instructed target numbers should be cancelled without omission. The primary measure of this test was the number of hits (correct answers). We used only the number of hits in the first trial.

***Cd*:** Digit Symbol Coding (Cd) was a subtest of WAIS-III [11]. This test measured processing speed. For Cd, the participants were shown a series of symbols that were paired with numbers. Using a key within a 120 s time limit, participants draw each symbol under its corresponding number. The primary measure of this test was the number of correct answers.

***SS*:** Symbol Search (SS), a subtest of WAIS-III containing 60 items [11], measured processing speed. For this subtest, participants visually scanned two groups of symbols (a target group and a search group) and reported whether either of the target symbols matches any symbol in the search group. Participants responded to as many items as possible within a 120 s time limit. The primary measure of this test was the number of correct answers.

References

1. Nouchi R, Taki Y, Takeuchi H, Hashizume H, Nozawa T, Sekiguchi A et al. Beneficial effects of reading aloud and solving simple arithmetic calculations (learning therapy) on a wide range of cognitive functions in the healthy elderly: study protocol for a randomized controlled trial. Trials. 2012;13:32. doi:10.1186/1745-6215-13-32.
2. Uchida S, Kawashima R. Reading and solving arithmetic problems improves cognitive functions of normal aged people: a randomized controlled study. Age (Dordr). 2008;30(1):21-9. doi:10.1007/s11357-007-9044-x.
3. Alvarez JA, Emory E. Executive function and the frontal lobes: a meta-analytic review. Neuropsychological Review. 2006;16(1):17-42. doi:10.1007/s11065-006-9002-x.
4. Hakoda Y, Sasaki M. Group version of the Stroop and reverse-Stroop test: the effects of reaction mode, order and practice. Kyoiku Shinrigaku Kenkyu (JPN J Educ Psychol). 1990;38:389-94.
5. Watanabe M, Hakoda Y, Matsumoto A. Group Version of the Stroop and Reverse-Stroop Test : An Asymmetric developmental trait in two kinds of interference. Kyusyu Daigaku Shinrigaku Kenkyu (Kyushu University Psychological Research) 2011;12:41-50.
6. Faul F, Erdfelder E, Buchner A, Lang AG. Statistical power analyses using G*Power 3.1: tests for correlation and regression analyses. Behavior Research Methods. 2009;41(4):1149-60. doi:10.3758/brm.41.4.1149.
7. Matsuoka K, Uno M, Kasai K, Koyama K, Kim Y. Estimation of premorbid IQ in individuals with Alzheimer's disease using Japanese ideographic script (Kanji) compound words: Japanese version of National Adult Reading Test. Psychiatry and Clinical Neurosciences. 2006;60(3):332-9. doi:10.1111/j.1440-1819.2006.01510.x.
8. Ito E, Hatta T, Ito Y, Kogure T, Watanabe H. Performance of verbal fluency tasks in Japanese healthy adults : Effect of gender, age and education on the performance. Shinkei Shinrigaku Kenkyu (JPN J Neuropsychol). 2004;20(4):254-63.
9. Wechsler DA. Wechsler Memory Scale Revised. San Antonio, TX.: The Psychological Corporation; 1987.
10. Wilson BA, Cockburn J, Baddeley AD. The Rivermead Behavioral Memory Test. Reading, England: Thamas Valley Test Company; 1985.
11. Wechsler DA. Wechsler Adult Intelligence Scale Third edition. San Antonio, TX.: The Psychological Corporation; 1997.
12. Matsuoka K, Uno M, Kasai K, Koyama K, Kim Y. Estimation of premorbid IQ in individuals with Alzheimer's disease using Japanese ideographic script (Kanji) compound words: Japanese version of National Adult Reading Test. Psychiatry Clin Neurosci. 2006;60(3):332-9. doi:10.1111/j.1440-1819.2006.01510.x.
13. Nelson HE. National Adult Reading Test (NART). Windsor, UK: NFER-Nelson; 1982.
14. Hatta T, Ito Y, Yoshizaki K. D-CAT manual (Screening test for attention). Osaka.: Union Press; 2000.
